# Supplementary material for: Enhancing titres of therapeutic viral vectors using the transgene repression in vector production (TRiP) system
Source: Nat Commun. 2017 Mar 27;8:14834. doi: 10.1038/ncomms14834 (PMC5378976; doi:10.1038/ncomms14834)
Supplement: Supplementary Information — Supplementary Figures, Supplementary Methods and Supplementary References [file ncomms14834-s1.pdf]

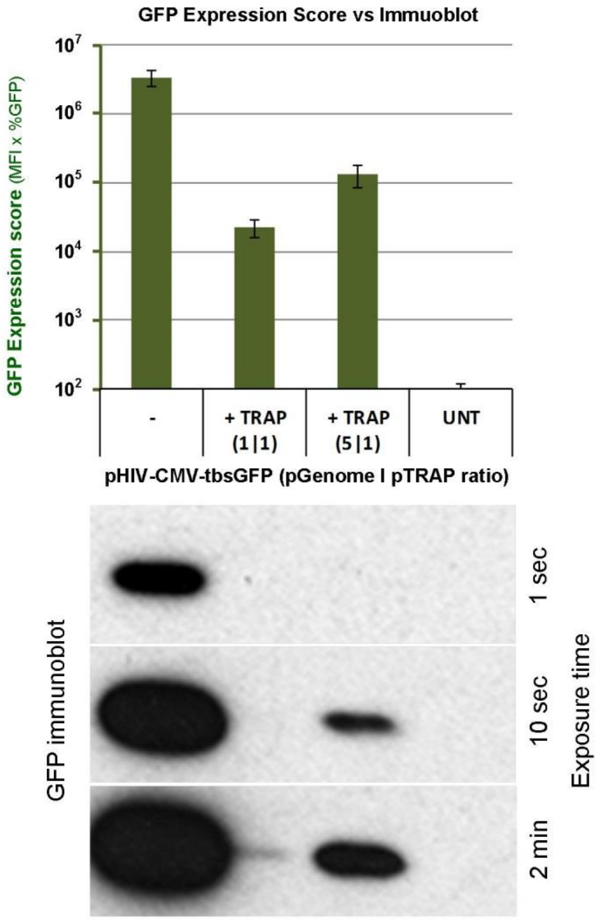

Supplementary figure 1. Comparison of GFP Expression Score and detection of GFP by immunoblotting. HEK293T cells were cotransfected with the GFP vector genome pHIV-CMV-tbsGFP at the stated molar ratios of pEF1a-coTRAP[H6] or pBluescript (-), and cells analysed by flow cytometry 2 days post-transfection. GFP Expression Scores (arbitrary units) used in the study were generated by multiplying the % GFP-positive cell number by the median fluorescence intensity (MFI). We applied this score throughout the study, as we found that measures of GFP expression based solely on MFI were often subject to skewing due to a small number of sporadic events (less than 1%) in GFP-positive gates, which presumably reflect cells that receive GFP reporter plasmid but not TRAP plasmid. In parallel, replicate cultures were lysed and cytoplasmic fractions analysed by immunoblotting (anti-GFP), which supports the suitability of the GFP Expression score to approximate GFP protein expression in cell cultures. All data are mean average values ± s.d. [$\log_{10}$-transformed data] (n=3). Data are representative of two independent experiments.

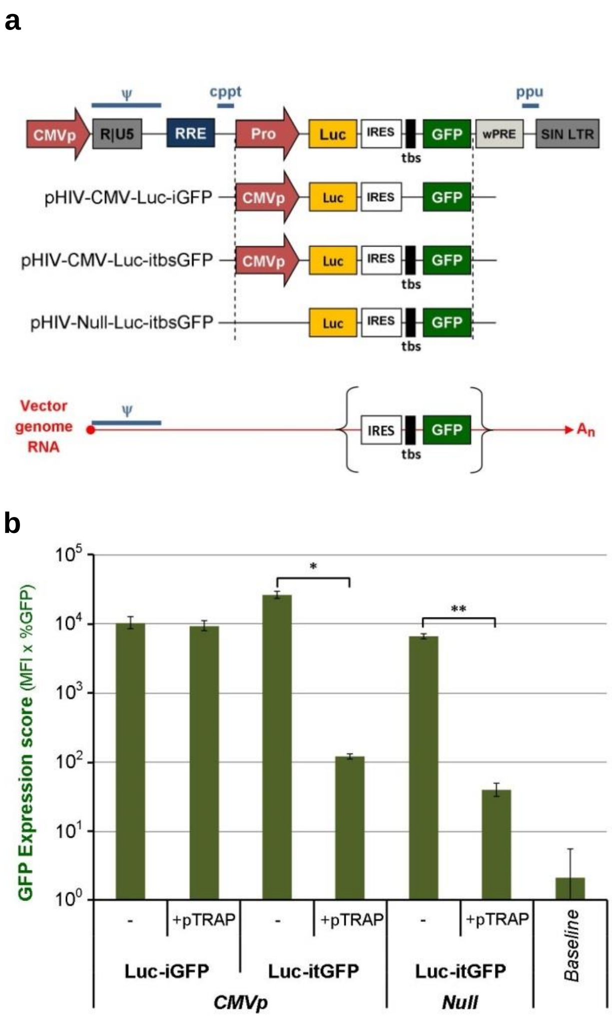

Supplementary figure 2. Expression of IRES-driven transgene expression from lentiviral vector genome RNA can be repressed by the TRiP system. (a) Bicistronic HIV-1 based vector genome plasmids were constructed wherein the internal transgene cassette (driven by either CMV promoter or having no promoter [Null]) contained a Luciferase-IRES-GFP reporter and the GFP gene at ORF2 was or was not TRAP-tbs controlled. Note that full length vector genome RNA molecules containing IRES-dependent ORFs are theoretically capable of transgene protein expression. (b) HEK293T cells were transfected with the bicistronic vector genomes with or without TRAP plasmid and cells analysed by flow cytometry 2 days post-transfection to generate GFP Expression Scores. Note the high level of GFP expression from pHIV-Null-Luc-itbsGFP (i.e. derived from vector genome RNA) could be repressed by TRAP. Data are mean average values ± s.d. [log_{10}-transformed data] (n=3); *$p<1.2x10^{-6}$, **$p<1.0x10^{-4}$.

(CMVp, Cytomegalovirus promoter; Ψ, packaging signal; RRE, rev-responsive element; cppt, central polypurine tract; Int Pro, internal promoter; tbs, TRAP-binding sequence; IRES, internal ribosomal entry site (encephalomyocarditis virus); wPRE, post-transcriptional regulatory element from Woodchuck Hepatitis Virus; ppu, polypurine tract; SIN, self-inactivating; A_n, poly-adenines). Data are representative of two independent experiments.

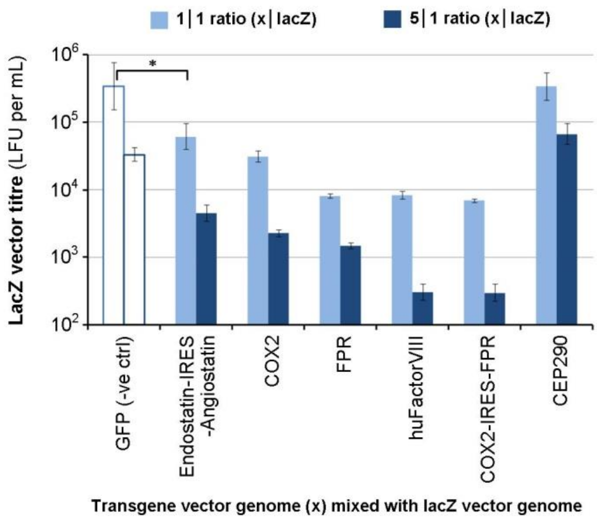

Supplementary figure 3. Assessment of therapeutic transgene protein expression on vector titres by vector genome mixing. The impact of the transgene protein on vector titres during production can be assessed by a simple mixing experiment. A reporter-encoding vector genome such as lacZ is mixed with therapeutic vector genome at the stated mass ratios of plasmid at transfection. The impact of transgene protein expression during vector production can be measured by reporter (lacZ) vector titration. Note that whilst GFP is considered to be inert, the effect of mixing a large proportion of GFP vector genome relative to lacZ (5:1) has the impact of reducing lacZ vector titres merely because proportionally more GFP-encoding vector particles are produced due to greater abundance of this vector genome RNA in the cell (a non-packageable therapeutic transgene expression plasmid can be used instead to minimise this effect). Therefore, data should only compared to GFP (no fill; negative control) at the same relative vector genome ratios. All data presented are from EIAV-based vectors: Endostatin and Angiostatin are encoded by RetinoStat®; COX2 and FPR are transgenes encoded within experimental vectors for Glaucoma treatment; Factor VIII is encoded within ReQuinate; CEP290 is encoded within experimental vectors for treatment of Leber congenital amauorsis. All data are mean average values ± s.d. [log_{10}-transformed data] (n=4); *$p<1.6x10^{-2}$. Data are representative of two independent experiments.

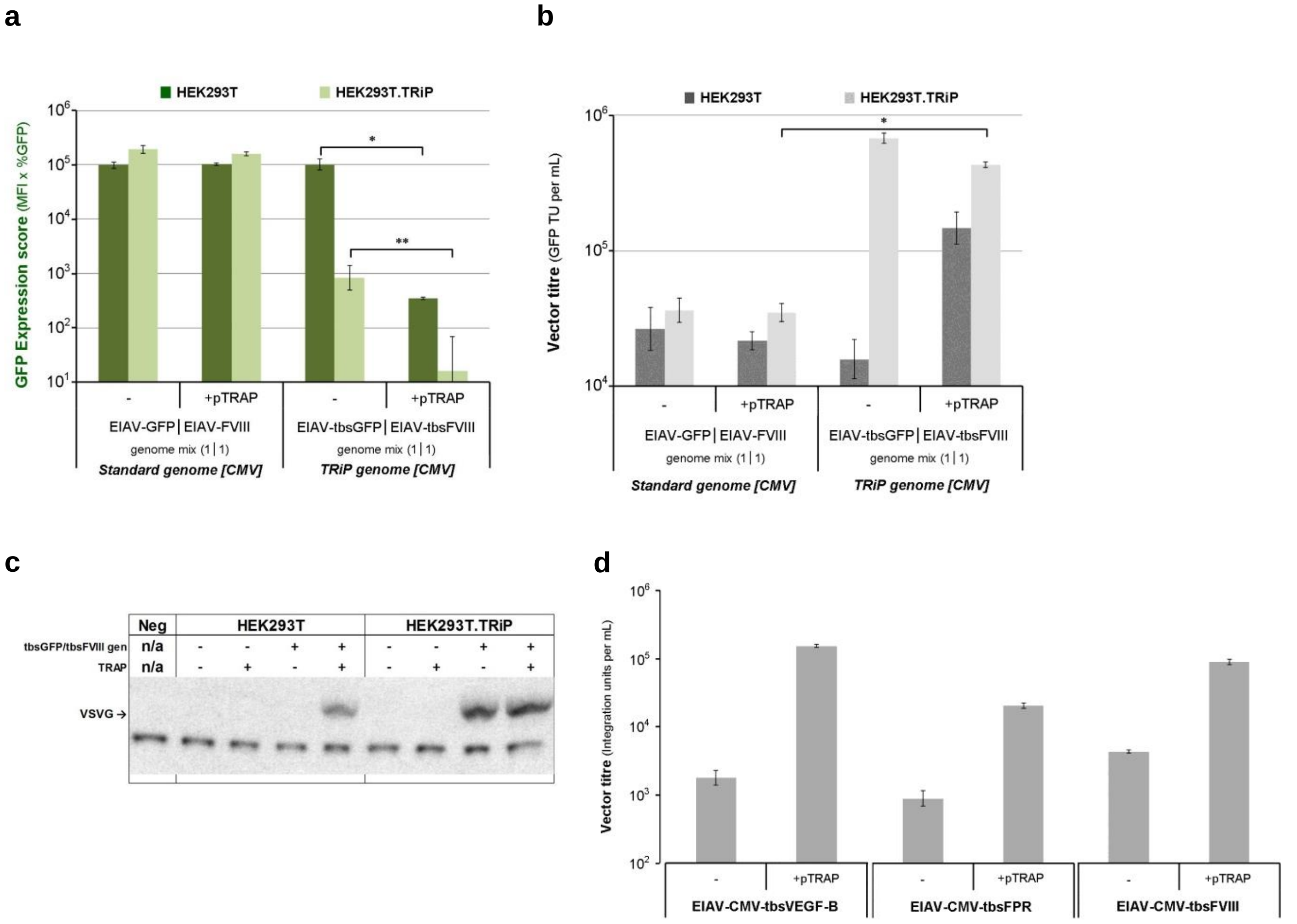

Supplementary figure 4. Comparison of 'transient' and 'stable' versions of the TRiPLenti system by vector genome mixing experiment. HEK293T cells and HEK293T.TRiPcells (stably expressing TRAP[H6]) were used to produce mixed EIAV-based vectors. Under 'standard' vector production protocol conditions, control vector genomes encoding either GFP or human Factor VIII were mixed 1:1 (mass ratio) prior to co-transfection of HEK293T cells together with EIAV vector packaging components, and either TRAP plasmid (+TRAP) or pBluescript (-). In the TRiP system, tbs-containing vector genomes encoding either GFP or Factor VIII were mixed 1:1 (mass ratio) prior to co-transfection of cells with packaging components. (a) Two days post-transfection, replicate cultures were analysed by flow cytometry to generate GFP Expression Scores for each condition i.e. to measure the direct effect of TRAP on GFP expression, and indirectly as a model for Factor VIII expression (note we were unable to develop a robust assay for human Factor VIII detection). Data are mean average values ± s.d. [$\log_{10}$-transformed data] (n=4); $*p<8.4x10^{-6}$, $**p<3.4x10^{-2}$. (b) Crude vector harvests generated were titrated by GFP transduction assay in HEK293T cells to measure impact of Factor VIII expression on vector titres. Data are mean average values ± s.d. [$\log_{10}$-transformed data] (n=4); $*p<1.8x10^{-5}$. (c) 50-fold concentrated vector preparations made in the stated cell lines were analysed by immunoblot for VSVG content (loading normalised by F-PERT assay); this is an indirect measure of Factor VIII activity as Factor VIII has been shown to inhibit VSVG incorporation into EIAV-based vector virions[1]. We have previously shown that subtle changes in VSVG pseudotyping can affect virion activity[2], and this observation supports the notion that pre-existing pools of TRAP protein can further benefit vector production in the TRiPLenti system, particularly when the impact of transgene protein is potent. (d) Enhanced titres of other Lentiviral (EIAV) vector genomes encoding biologically active transgenes, produced using the TRIPLenti system; VEGF-B (n=4), FPR (Prostaglandin receptor) (n=4), human Factor VIII (n=2). Data are mean average values ± s.d. [$\log_{10}$-transformed data].

a

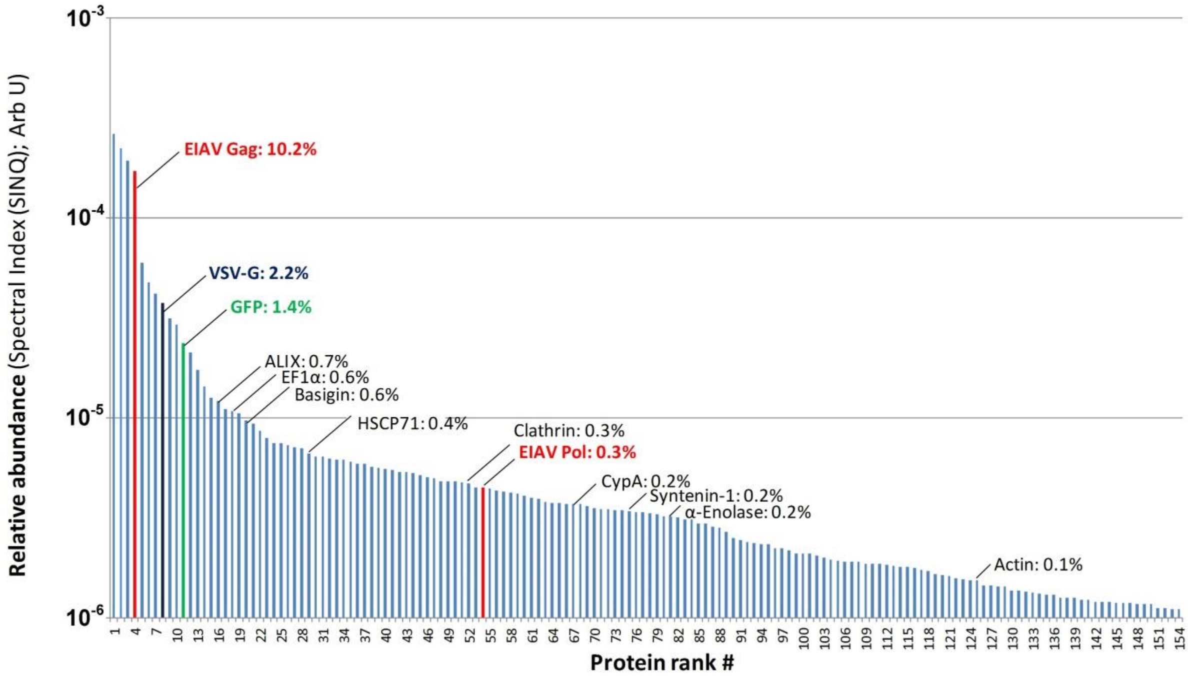

b

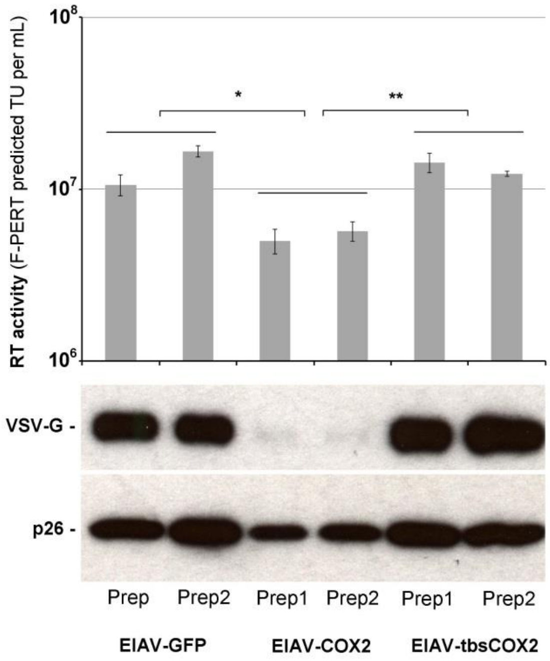

Supplementary figure 5. Protein profiling of EIAV vector preps using SINQ. (a) A selection of protein hits in the top 154 proteins identified in duplicate EIAV-GFP vector preps to assess data quality. Only proteins that varied less than 4-fold between duplicate samples are included, and proteins of note are labelled with percentage proportion, which was generated by dividing the average Spectral index scores for each hit by the sum of all scores in the top 154 hits. EIAV Gag and Pol peptides appeared were detected at expected ratios, and VSVG/GFP were present in high abundance. Selected cellular proteins known to be incorporated into HIV-1 virions are highlighted. (b) Concentrated EIAV vector preps used for SINQ analysis were analysed by F-PERT (RT activity) and titrated against a standard EIAV-GFP prep of known biological activity to yield an arbitrary evaluation of titre (F-PERT-predicted titre), and also analysed by SDS-PAGE/Immunoblotting to EIAV capsid (p26) and VSV-G. Data are mean average values ± s.d. [log_{10}-transformed data] (n=4); *$p<2.9x10^{-3}$, **$p<7.5x10^{-5}$. Data are representative of two independent experiments.

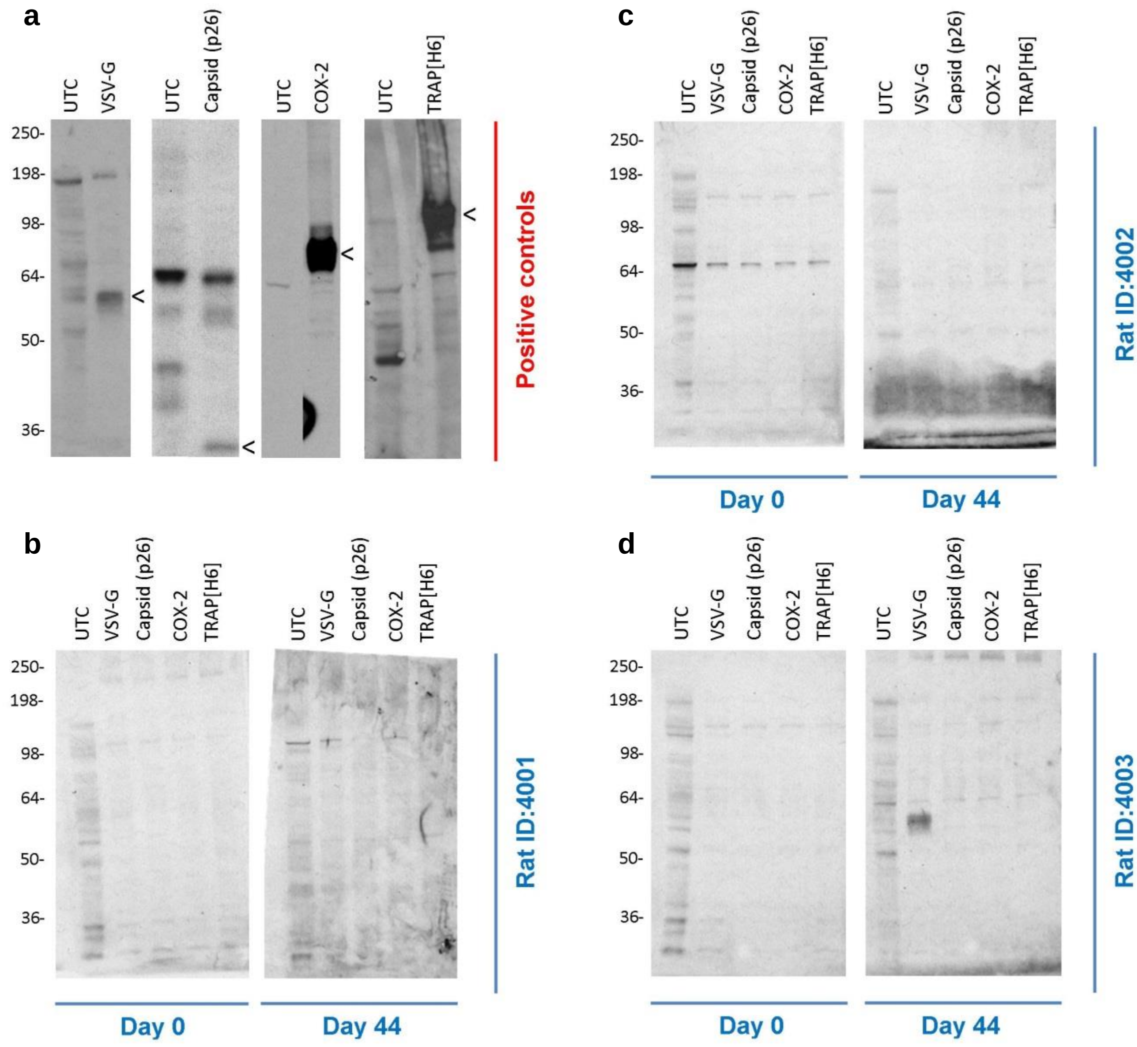

Supplementary figure 6. Analysis of rat sera post-intracameral treatment with EIAV-tbsCOX2. As part of an investigation of a gene therapy for primary open-angle glaucoma, Wistar Hannover rats were injected with ~$1x10^4$ TUs of concentrated EIAV-tbsCOX2 into the front anterior eye chamber and assessed for inflammatory signals (this route of administration is extremely sensitive to inflammation), as well as immunological response to vector constituents. This was achieved by using sera from Day 0 or Day 44 post-injection as primary antibody to immunoblots of non-denaturing SDS-PAGE separated HEK293T cell lysates expressing individual vector components. (a) Positive control immunoblots indicating specific bands to VSV-G, EIAV Capsid (p26), COX2 (blot lane re-positioned relative to UTC for clarity) and TRAP. Note that under non-denaturing conditions the intact TRAP 11-mer is detected. (c-d) Tests performed on sera from three animals, indicating no immune response to vector components including TRAP[H6], except in one animal (4003) where antibodies to VSV-G were present at Day 44. All blots were exposed for the same amount of time. Vector was well-tolerated in this study with no vector-related inflammation and most ocular changes were secondary to the intracameral dosing procedure alone (data not shown). There were no treatment-related clinical signs or ophthalmic findings following dosing in any animal with vector in this study (data not shown). This is encouraging data that indicates the presence of TRAP in vector preps does not increase vector immunogenicity.

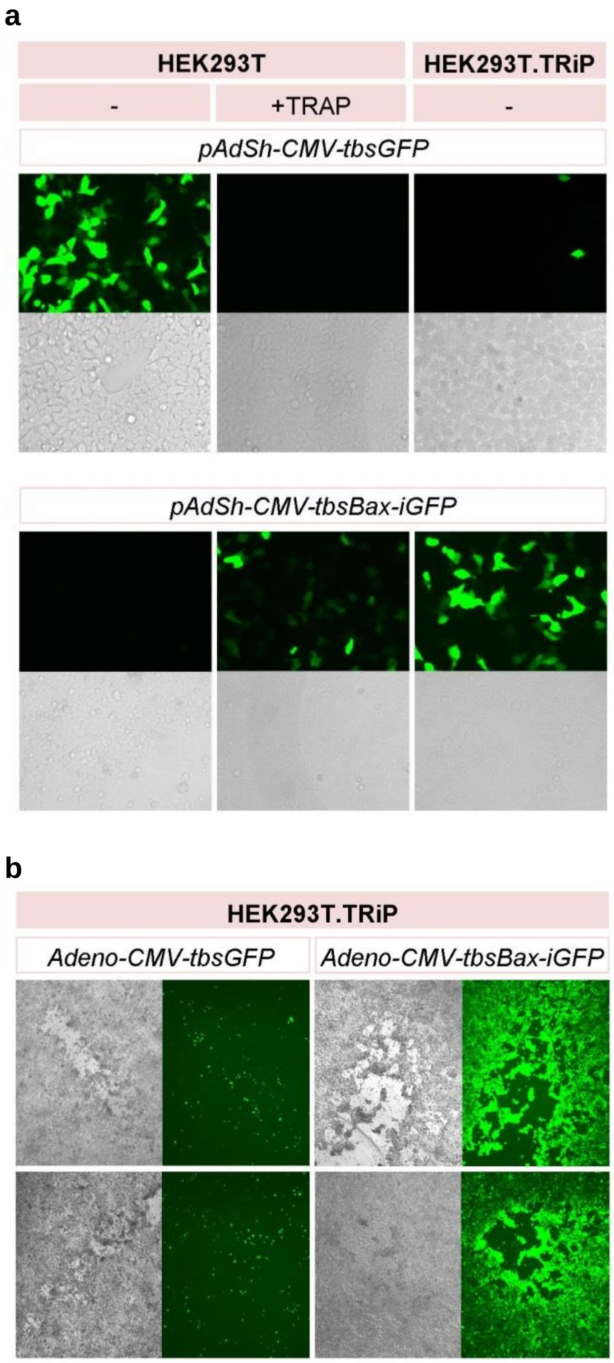

Supplementary figure 7. Images of cultures of HEK293T or HEK293T.TRiP cells under fluorescence during the recombination steps of Adeno-CMV-tbsGFP and Adeno-CMV-tbsBax-iGFP. (a) Representative images from triplicate cultures two days post-transfection with the indicated shuttle plasmids together with the pacAd5 9.2-100 Adenoviral vector backbone. The GFP expression in cells transfected with pAdShuttle-CMV-tbsGFP was inversely linked to the presence of TRAP expressed within the cells. Conversely, GFP expression in cells transfected with pAdShuttle-CMV-tbsBax-iGFP was only apparent in cells expressing TRAP indicating that expression of Bax within HEK293T cells impacts on protein expression, presumably due to induction of apoptosis. These cultures were incubated for 14 days to allow for plaque formation. (b) Representative images of triplicate HEK293T.TRiP cultures 12 days post-transfection with Adenoviral vector components. High level of GFP was observed within cultures producing Adeno-CMV-tbsBax-iGFP compared to Adeno-CMV-tbsGFP, and the formation of plaques with Adeno-CMV-tbsBax-iGFP was associated with higher GFP levels compared to the surrounding monolayers of cells. This gave an early indication that Adeno-CMV-tbsBax-iGFP had successfully recombined within HEK293T.TRiP cells. No plaques were detected within HEK293T cultures with either vector type by day 15 post-transfection.

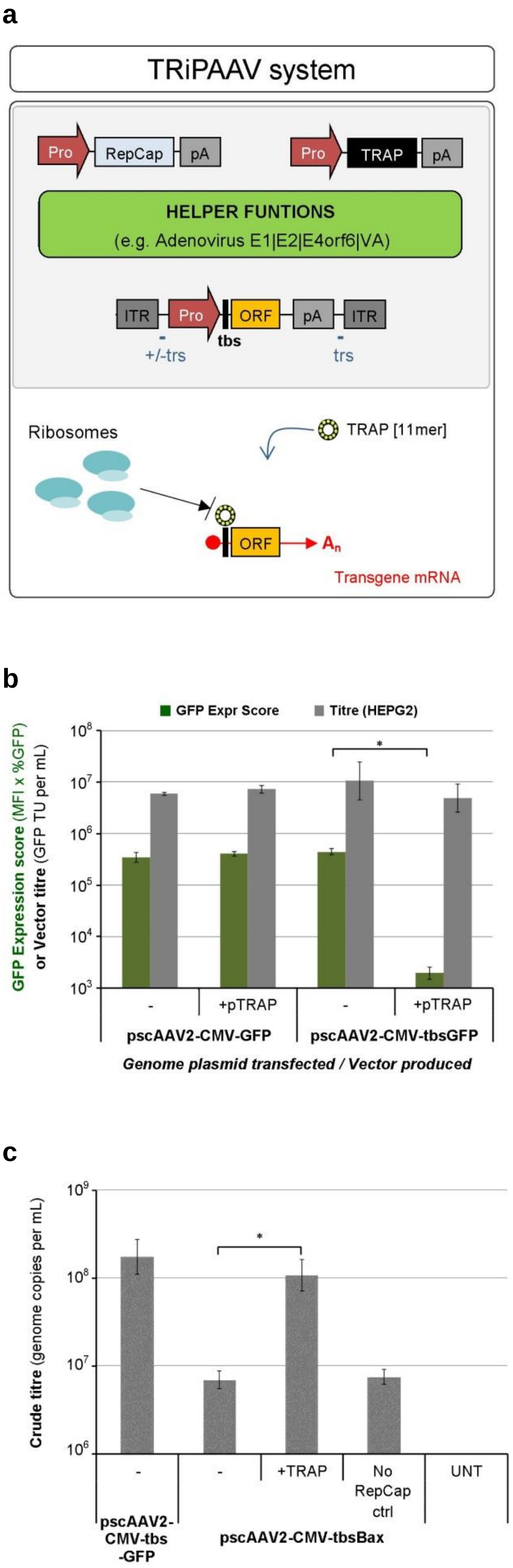

Supplementary figure 8. Exemplifcation of the TRiPAAV system. (a) The TRiPAAV system, which requires: vector genome (with tbs-modified transgene; typically a single transgene, although small TRAP-tbs controlled bicistronic cassettes are theoretically possible with 5kb genomes), rep-cap, helper functions (e.g. Adenovirus regulatory components) and a TRAP expression cassette. (b) Plasmids encoding AAV2-CMV-GFP and AAV2-CMV-tbsGFP were each co-transfected with pRepCap, and either TRAP plasmid or pBluescript (-) into HEK293T cells. GFP Expression Scores (green bars) were generated by flow cytometry of replicate cultures 3 days post-transfection, and titres (HEPG2 cells; grey bars) of purified vector particles generated. Data are mean average values ± s.d. [log_{10}-transformed data] (n=3); *$p<9.9x10^{-5}$. (c) Production of scAAV2-CMV-tbsGFP in HEK293T cells was repeated, together with scAAV2-CMV-tbsBax, which encodes the pro-apoptotic gene Bax. vDNA in crude vector material was quantified by qPCR using a primer/probe set to CMVp (only vector genome plasmid contained a CMV promoter). A negative control transfection was included (No RepCap) to control for residual pDNA within crude harvest material, which was substantial despite our efforts to reduce DNA using Benzonase®. Nevertheless, we were able to detect scAAV-CMV-tbsBax vDNA at similar levels to scAAV-CMV-tbsGFP, only when TRAP plasmid was supplied during transfection. Data are mean average values ± s.d. [log_{10}-transformed data] (n=3); *$p<6.0x10^{-7}$. (Pro, promoter; ITR, inverted terminal repeat; trs, terminal resolution site; tbs, TRAP-binding sequence; ORF, open-reading frame; polyA, polyadenylation signal; An, poly-adenines).

Supplementary figure 9. Full blot images of exposures of immunoblot displayed in Figure 4e of the main report (see main report experiment details). (a) Cell lysates probed against COX-2, 8 hours post-transfection. (b) Cell lysates probed against COX-2, 32 hours post-transfection. (c) End-of-integration assay cell lysates (10 days post-transduction) probed against COX-2.

a

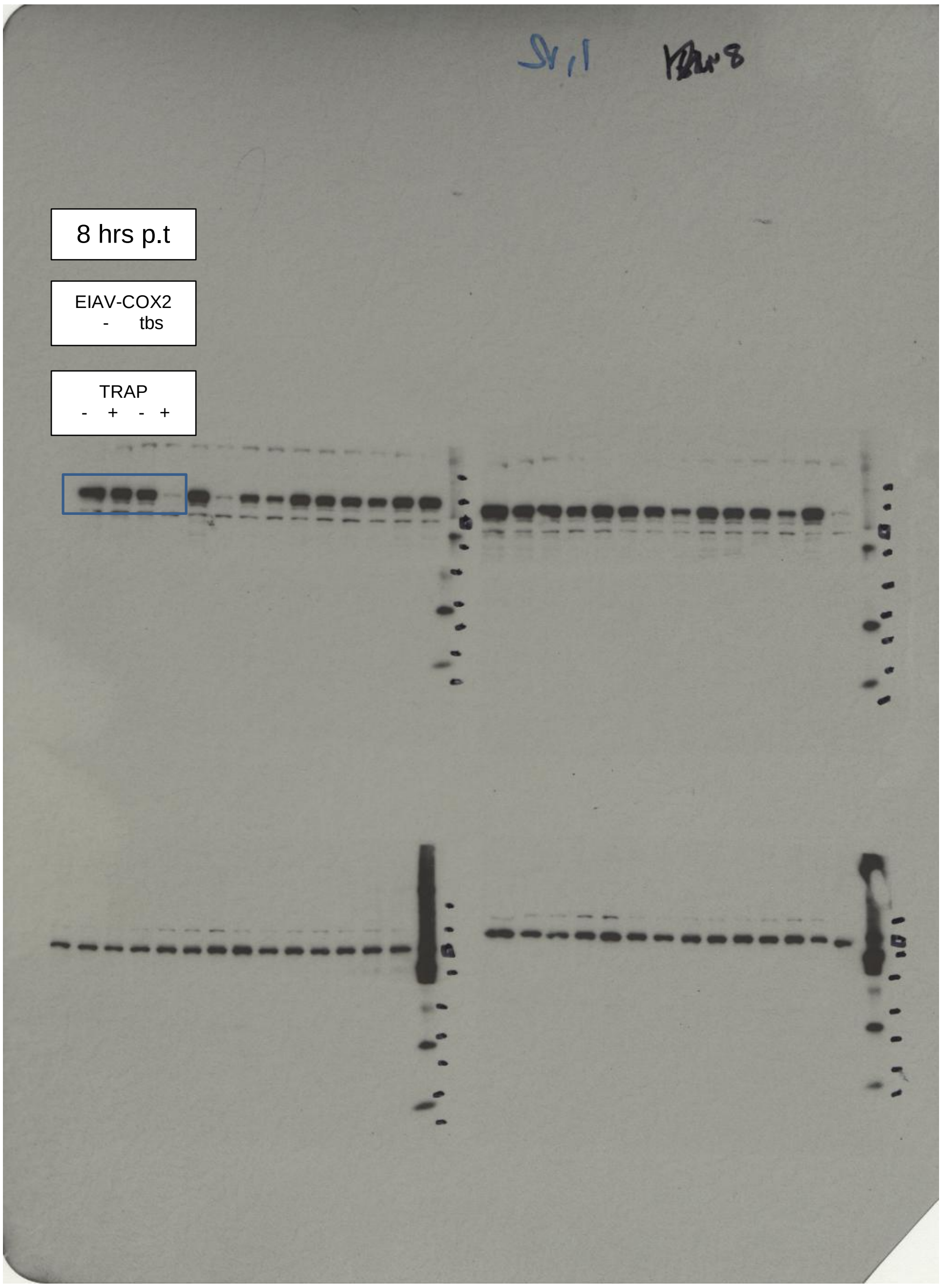

b

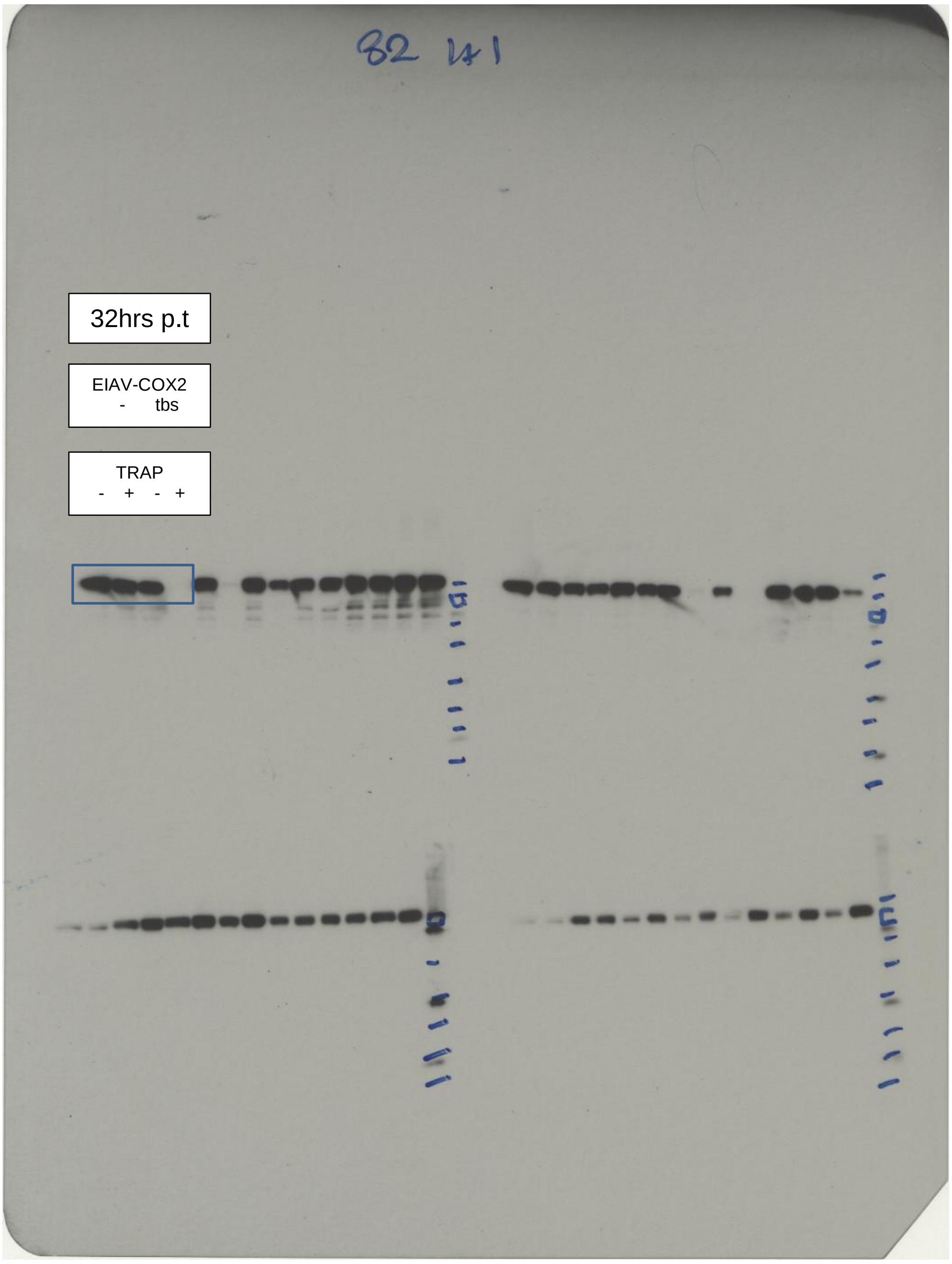

c

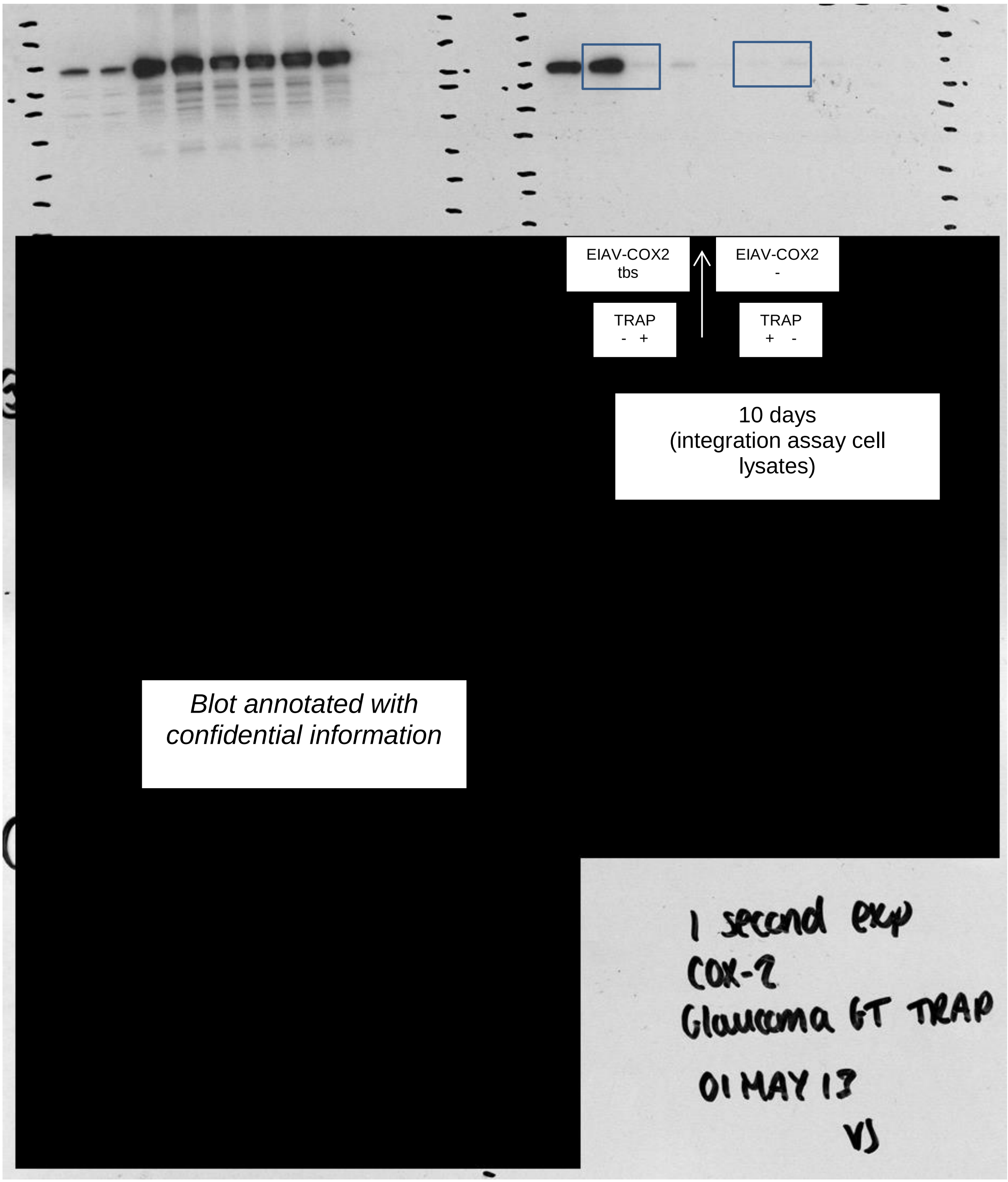
EIAV-COX2
tbs
EIAV-COX2
-
TRAP
- +
TRAP
+ -
10 days
(integration assay cell lysates)
[redacted]
Blot annotated with confidential information
1 second exp
COX-2
Glaucoma GT TRAP
01 MAY 13
VJ

Supplementary figure 10. Full blot images of exposures of immunoblot displayed in Figure 7d of the main report (see main report experiment details). Western blots were cut at two positions (between 19kDa and 26kDa, and between 26kDa and 37kDa markers; see arrows) and probed for GFP (~27kDa), Bax (~21kDa), cleaved Poly (ADP-ribose) polymerase (clv-PARP; ~89kDa), and GAPDH (~37kDa). The separate blots were re-aligned during ECL/film development.

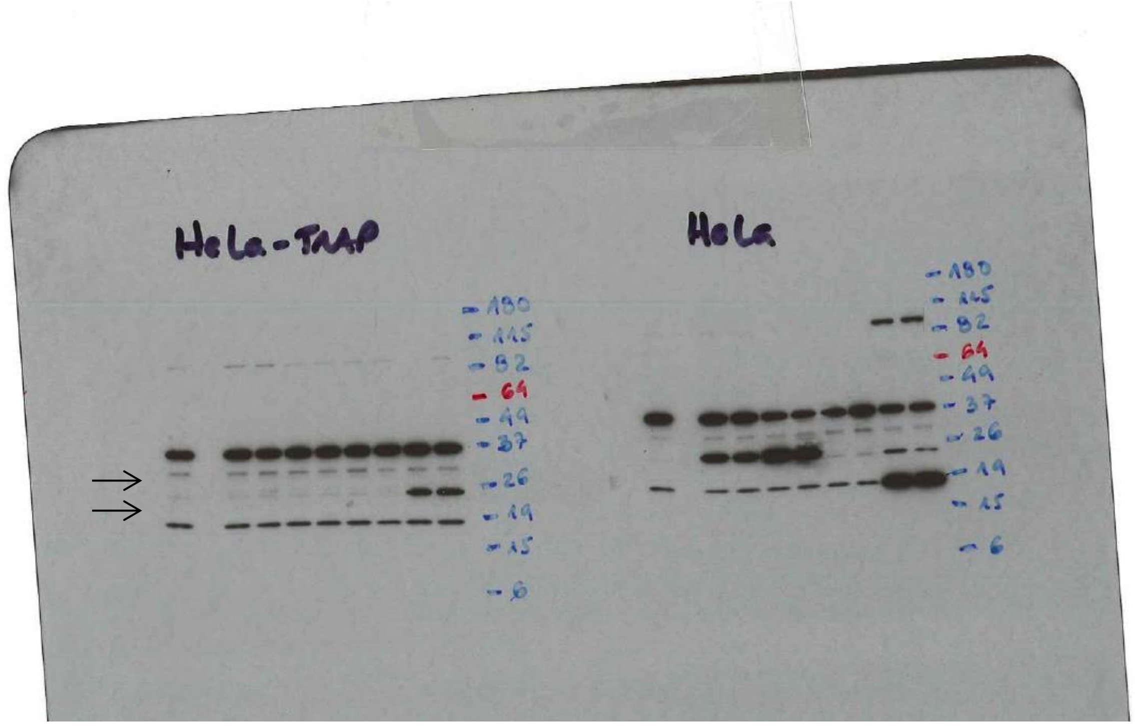

Supplementary methods

Evaluation of the methods of GFP protein expression in transfected HEK293T cells

HEK293T cells were transfected with pHIV-CMV-GFP or pHIV-CMV-tbsGFP vector genome plasmids +/- pEF1a-coTRAP[H6]. Expression of GFP was carried out by flow cytometry (FACSVerse, BD Biosciences), out-gating dead cells prior to analysis of FL1 channel events. GFP Expression scores were calculated by multiplying % GFP-positive cells by the median fluorescence intensity of events within the % GFP-positive gate. GFP expression in cell lysates was carried out by SDS-PAGE under reducing conditions, Western transfer and immunoblotting using antibodies to GFP (Ab290, AbCam). Species-specific HRP conjugated secondary antibodies were used at 1:1000 dilution.

Testing the level of IRES-dependent transgene expression from HIV-1-based vector genome plasmid DNA in HEK293T cells

HEK293T cells were transfected with pHIV-Luc-IRES-GFP based vector genome plasmids +/- pEF1a-coTRAP[H6]. Vector genomes either contained an internal CMV promoter or no promoter, and were differentially controlled by TRAP/tbs at the ORF2 positions, encoding GFP. Expression of GFP was carried out by flow cytometry (FACSVerse, BD Biosciences), out-gating dead cells prior to analysis of FL1 channel events. GFP Expression scores were calculated by multiplying % GFP-positive cells by the median fluorescence intensity of events within the % GFP-positive gate.

Evaluation of impact of transgene by vector genome plasmid mixing experiments

The impact of therapeutic transgene encoded protein on vector titres was carried out by a vector genome plasmid mixing experiment. Therapeutic EIAV vector genome plasmid was mixed at either 1:1 or 5:1 mass ratio with an EIAV-lacZ vector genome plasmid during transfection of vector components. EIAV-GFP was used as a negative control using the same genome plasmid ratios so that lacZ genome dilution could be accounted for. Crude vector was generated as stated in Materials and Methods, and titrated by serial 10-fold dilution followed by transduction of D17 cells in the presence of 8µg per mL polybrene for 5-6 hours. Fresh media was added before a 3 day incubation, followed by fixation in formalin and staining with 0.04% X-Gal. Transducing events (2 or more clusters of blue cells) were counted, from which lacZ TU per mL titres were calculated. The lacZ titres generated for each mix with therapeutic vector genome must be compared to the equivalent GFP|lacZ control mix in order to evaluate the impact of transgene on vector titres.

To assess the benefits of the stable TRiP system over the transient TRiP system, EIAV vector genome plasmids encoding human Factor VIII or GFP were mixed during production. HEK293T cells and HEK293T.TRiP cells (stably expressing TRAP[H6]) were used to produce mixed EIAV-based vectors. Under 'standard' vector production protocol conditions, control vector genomes encoding either GFP or human Factor VIII were mixed 1:1 (mass ratio) prior to transfection of HEK293T cells together with EIAV vector packaging components, and either TRAP plasmid (+TRAP) or pBluescript (-). In the TRiP system, tbs-containing vector genomes encoding either GFP or human Factor VIII were mixed 1:1 (mass ratio) prior to transfection of HEK293T.TRiP cells together with EIAV vector packaging components and either TRAP plasmid (+TRAP) or pBluescript (-). Two days post-transfection, replicate cultures were analysed by flow cytometry to generate GFP Expression scores for each condition i.e. to measure the direct effect of TRAP on GFP expression, and indirectly as a model for human Factor VIII expression (note we were unable to develop a robust assay for human Factor VIII detection). Crude vector harvests generated were titrated by GFP transduction assay in HEK293T cells to measure impact of human Factor VIII expression on vector titres. 50-fold concentrated vector virions were analysed by immunoblot for VSVG content; an indirect measure of human Factor VIII activity (human Factor VIII has been shown to inhibit VSVG incorporation into EIAV-based vector virions[14]).

Non-SINQ analysis of EIAV-GFP, EIAV-COX2 and EIAV-tbsCOX2 vector preparations

Physical titration of concentrated EIAV vector particles was carried out by F-PERT[3] and SDS-PAGE was carried out on samples by loading either equal volumes or by F-PERT-normalised volumes. Immunoblotting was carried out using anti-VSV-G (AFPA1-30138, Fisher) and anti-EIAV p26 (capsid; mAb 12E8.1) antibodies. Species-specific HRP conjugated secondary antibodies were used at 1:1000 dilution.

Testing putative antibody response to EIAV-tbsCOX2 vectors produced using the TRiPLenti system in vivo by rat serum analysis

A volume containing approximately 10^4 transducing units (HEK293T DNA integration assay units) of ~2000-fold concentrated (double centrifugation) EIAV-tbsCOX2 vector was delivered into the front of the anterior chamber (intracamerally) of Wistar Hannover rat eyes (Crl:WI(Han); male, ~7 weeks old at time of dosing). This was part of a 56 day study to investigate gene transfer as part of the development of a gene therapy for primary open-angle glaucoma. Serum samples at day 0 and 44 were analysed by use as primary antibody samples (1:200 dilution) against Western blots. Western blots were generated by non-denaturing SDS-PAGE

and transfer of protein from HEK293T cell cultures individually expressing VSV-G or EIAV GagPol(p26) or COX-2 or TRAP[H6]. Primary antibodies [dilutions] used as positive controls were used for detection of VSVG (AFPA1-30138, Fisher) [1:1000], EIAV p26 (EIAVP6A1, VMRD) [1:200], COX-2 (CAY160112, Cayman Chemical) [1:1000], and TRAP[H6] (PA1-983B, Pierce) [1:1000]. Species-specific HRP conjugated secondary antibodies were used at 1:1000 dilution. *The total number of animals used in this study was considered the minimum required to properly characterise the effects of the test articles. The study was designed such that it did not require an unnecessary number of animals to accomplish its objectives. This study was not conducted in compliance with GLP regulations; however, it was conducted using good scientific practices, following the applicable SOPs and under the ethical regulations of the Test Facility (Charles River Laboratories, Preclinical Services, Montreal (PCS-MTL), 22022 Transcanadienne, Senneville, QC H9X 3R3, Canada).*

Production and titration of scAAV-based vectors

For small scale production of scAAV vectors, HEK293T cells were seeded at $4.2x10^6$ cell per plate in 10mL complete HEK293T media and incubated at 37ºC in 5% CO2 through-out production. Approximately 20 hours later, cells were transfected using the following mass ratios of plasmids for AAV vector production: 2µg genome, 2µg pRepCap2, 2µg pHelper, and 2µg TRAP or pBlueScript. This represented a genome:TRAP plasmid molar ratio of 1:1. Transfection was mediated using Lipofectamine® 2000CD and Opti-MEM® in the ratios stated above. Approximately 24 hours later, 10mL fresh complete media replaced the transfection media and cultured for ~54 hours, before cells were harvested. AAV virions were purified using a Virabind™ kit (Cellbiolabs), resulting in 100µL concentrated preparations in PBS. For repeated scAAV-CMV-tbsGFP and scAAV-CMV-tbsBax production, the above methods were scaled down to 6-well scale by vessel area. Pelleted cells were freeze-thawed 3-4 times generating 1mL crude vector stocks.

Titration of GFP-encoding vectors occurred by serial dilution and 5-6 hour transduction of HEPG2 cells (ECACC, 85011430) in serum-free before addition of fresh media, followed by incubation for 2 days. Target cell counts were made prior to transduction. Cultures were analysed for percent GFP expression using a FACSVerse™ and vector titres calculated accordingly. For qPCR of scAAV vectors, 0.25mL of crude vector preps were treated as previously published[4], and resulting material diluted 100-fold and inoculated into qPCR reactions comprised of Taqman® Universal PCR master mix (Life Technologies) under standard chemistry PCR cycling conditions using a QuantStudio™ 6 (Life Technologies). A primer-FAM-probe set was designed to specifically detect a region of the CMV ORF:

Fwd - 5’·CATATATGGAGTTCCGCGTTACAT·3’

Probe - 5’·[FAM]TGGCTGACCGCCCAACGACC[TAMRA]·3’

Rev- 5’·CTATTGGCGTTACTATGGGAACATAC·3’

Statistical analysis

Statistical analysis was performed by Welch's unequal variances t-test (two-tailed, type 3) using log10-transformed data. $p<0.05$ was considered to be significant.

Supplementary references

1. Radcliffe, P.A. *et al.* Analysis of factor VIII mediated suppression of lentiviral vector titers. *Gene Ther*. 15, 289-297 (2008).

2. Farley, D.C. et al. Factors that influence VSV-G pseudotyping and transduction efficiency of lentiviral vectors-in vitro and in vivo implications. J. Gene Med. 9, 345-356 (2007).

3. Rohll, J.B. et al. Design, production, safety, evaluation, and clinical applications of nonprimate lentiviral vectors. Methods Enzymol. 346, 466-500 (2002).

4. Strobel, B, *et al*. Riboswitch-mediated Attenuation of Transgene Cytotoxicity Increases Adeno-associated Virus Vector Yields in HEK-293 Cells. *Mol. Ther* **23**, 1582–1591 (2015).
